# Editorial: Emerging contaminants in children: exposure, sources, and health effects

**DOI:** 10.3389/fped.2025.1659009

**Published:** 2025-09-01

**Authors:** Manoj Kumawat, Shiming Song, Chao Zhang

**Affiliations:** ^1^ Department of Biological Sciences, Indian Institute of Science Education and Research, Bhopal, India; ^2^ School of Environmental Science and Engineering, Sun Yat-sen University, Guangzhou, China; ^3^ SCNU Environmental Research Institute, South China Normal University, Guangzhou, China

**Keywords:** emerging contaminant (EC), children health, pollutants (environmental), environmental exposure, pollution

**Editorial on the Research Topic**
Emerging contaminants in children: exposure, sources, and health effects

Children are the next generation, and they will be the ones who represent today in the future. The physical and mental development of children is a significant phenomenon that reflects a healthy lifestyle. Children are growing up in an invisible threat these days, which can lead to immune system failure, endocrine system disruption, and developmental issues. Emerging contaminants (ECs) are a class of recently identified pollutants, chemicals, and materials that can be harmful to human health, especially in developing bodies. Emerging contaminants are newly recognised synthetic or naturally occurring chemical or biological agents found in the environment that may pose risks or have recently been identified as hazardous to human health and ecosystems (1, 2). As a result of human activity, the environments in which children live, learn, and play are becoming more contaminated. These environments include pesticides, industrial chemicals, personal care items, pharmaceuticals, and microplastics, which are increasingly present in water, soil, and the air and pose a risk to ecosystems and human health (3, 4).

The health of children is increasingly in danger from emerging pollutants, a concern that has grown over time as environmental scientists have concentrated more on identifying and reducing these risks. Pollutants were generally well defined in the past, but as consumer goods and industrialisation developed, so did the quantity and diversity of hazardous compounds (5). Compliant wood chip depths reduce impact forces, making it imperative to perform regular playground surface inspection and maintenance to safety standards to help prevent children's fall injuries. The identification and characterisation of these novel pollutants, many of which were not originally intended to interact with the human body, much less a child's developing physiology, has advanced significantly in recent years. At an alarming rate, scientists are already discovering these compounds in household items, food packaging, water, and air (6). Young ones, who explore their surroundings by crawling on the ground, tend to put items in their mouths and have a quicker breathing rate compared to adults, facing heightened risks. Paediatric exposure to specific toxicants poses a higher severe risk; enhancing safety regulations, education, and public health efforts remains crucial to protect children's health and prevent incidents. Since new chemical compounds are always being created and science is constantly expanding its knowledge of both present and historical pollutants, contaminants of emerging concern will remain a changing target. Proactive regulation, technological innovation, and thorough risk assessments are becoming increasingly necessary as this issue develops (7).

Pollution remains a critical global challenge, leading to millions of untimely fatalities each year. The advancement of new analytical techniques and technologies has greatly improved the detection and analysis of ECs. The health outcomes linked to these exposures present serious concerns; younger individuals are more susceptible to even minimal exposures, and the long-term risks are considerable, often irreversible (8). Airborne pollen, especially tree pollen, significantly impacts childhood asthma. Accounting for pollutants and weather strengthens this link. Targeted interventions, monitoring, and education can improve asthma management and reduce public health burdens. Recent investigations reveal distinct links between exposure to contaminants and an increase in developmental disorders, disruptions in the endocrine system, and dysfunctions in the immune system among children (9). Recent research found occupational pesticide exposure linked to leukaemia in 86% of studies, though study limitations hinder identifying specific agents. We need more research and public health policies to decrease exposure and cancer risk. There is an immediate necessity for enhanced strategies in risk assessment and management to effectively monitor and regulate emerging contaminants. It is essential to formulate policies that emphasise prevention rather than reaction, including stricter safety standards, regular exposure assessments, and prohibitions on the most hazardous substances.

The qualification of what is considered “emerging” is highly relative, particularly in the context of significant environmental contaminants that are currently gaining attention. It is promising to see that technological advancements are starting to enhance our capacity to identify and track these pollutants. Advanced analytical tools such as high-resolution mass spectrometry and biosensors are facilitating the identification of previously undetectable chemicals in samples from children's environments (7). The advancement of multi-omic methodologies, including genomics, proteomics, transcriptomics, and metabolomics, has facilitated the identification of molecular alterations induced by environmental exposure (10). Researchers are increasingly using these techniques to investigate how environmental contaminants affect the biological functions of organisms. These advancements are crucial for prompt identification and swift action.

Engaging with international environmental agencies related to ECs and participating in global initiatives for managing these contaminants is essential. It is critical to consider both community and global viewpoints. Children may experience heightened levels of exposure in communities with limited resources or marginalised communities, where environmental safeguards tend to be less stringent (11). The dissemination of contaminants across the globe transcends borders; what infiltrates one ecosystem frequently finds its way into another. This situation elevates the issue from a local matter to a global crisis affecting the environmental health of children.

Protecting children from new contaminants demands immediate partnership among researchers, policymakers, industry stakeholders, and the community. To address this challenge effectively, thorough investigation is essential for understanding the origins and possible impacts of these pollutants on children's health, ecosystems, and agricultural animals while embracing the one health approach. It is essential that we engage in informed action and foster innovation to ensure a healthy future for those who will come after us. When considering the well-being of children, any level of risk is simply unacceptable.
